# Lipid profile of different infant formulas for infants

**DOI:** 10.1371/journal.pone.0177812

**Published:** 2017-06-01

**Authors:** Marcio Antonio Mendonça, Wilma Maria Coelho Araújo, Luiz Antonio Borgo, Ernandes de Rodrigues Alencar

**Affiliations:** 1 Faculty of Agronomy and Veterinary Medicine, University of Brasilia, Brasilia, Brazil; 2 Department of Nutrition, College of Health Sciences, University of Brasilia, Brasilia, Brazil; University of Auckland, NEW ZEALAND

## Abstract

Situations including premature infants, or those in which there is a rejection to breastfeeding, require the use infant formulas for total or partial replacement of human milk. The objective of this study was to determine the lipid content and to identify the lipid profile of infant formulas. Samples were collected from ten different infant formulas, used as a substitute for breast milk at the Maternal and Child Hospital of Brasilia. The human milk sample consisted of a pool of samples from 10 mature milk donors at the milk bank of the University Hospital of Brasilia. The lipid content and lipid profile of the different infant formulas and human milk were analyzed. The experiment was conducted in a randomized block design, with eleven treatments and three replicates, in triplicate. The data obtained in this study indicated significant differences between infant formulas and human milk, and among the infant formulas analyzed in relation to the percentage of total lipids and the fatty acid profile, except for the fractions of linoleic acid and linolenic acid. Regarding the percentage of polyunsaturated fatty acids in relation to the total unsaturated fatty acids, only the Soy Protein Isolate-based Infant Formula (SPIIF) and Whey Protein Extensively Hydrolyzed Infant Formula (WPEHIF) resembled human milk. It was concluded that despite the observed differences, the use of infant formulas is a viable strategy for the development of infants subjected or not to specific physiological conditions.

## Introduction

Lipids of human milk are distinguished by their innumerable nutritional and physiological functions that favor infant development. About 98 g/100g of total lipids in human milk are triglyceride esters, and 90 g/100g of these are fatty acids. Thus, esterified fatty acids correspond to 88 g/100g of the lipid fraction [1,2,3]. These nutrients contain more than 200 fatty acids, and compared to bovine milk, contain a lower concentration of saturated fatty acids, a higher content of oleic and linoleic acids, a lower amount of other polyunsaturated fatty acids and lower hypercholesterolemic activity. They are sources of polyunsaturated fatty acids—including linoleic n-6 (18:2n-6) and α-linolenic n-3 acids (18:3n-3), both essential, and their derivatives 20:4n-6, 20:5n-3 and 22:6n-3, required for numerous physiological functions [1,4,5,6,7].

The total lipid content and fatty acid composition are variable, with positive modulation factors for adiposity, duration of the lactation period, lactation stage and maternal age, while maternal malnutrition, infections, metabolic disorders and medicines are considerable negative modulation factors, in addition to genetic factors, dietary habits, maternal diet, gestational age, hormones, parity and daily variation among lactations [8].

Specific conditions such as premature infants or those with contraindications to breastfeeding—maternal infection with the HIV virus, HTLV (human T cell lymphotropic virus), cytomegalovirus, herpes simplex or herpes zoster, chickenpox, hepatitis C, leprosy, Chagas disease and many others, determine the need for infant supplementation in addition to or in substitution of human milk [9,10,11]. In these situations, the safe alternative is the use of infant formulas because their composition is analogous to that of human milk, or they are suitable as a complete or partial substitute for breast milk [12].

In recent decades, many studies have been developed by infant formula manufacturers in an attempt to find nutritionally balanced and human milk-like formulations, especially with respect to the relationship between n-6 (C18:2 –linoleic) and n-3 fatty acids (C18:3 –linolenic) [12,13,14]. This is because essential fatty acids (EFAs) make up a class of molecules that cannot be synthesized by the body, due to lack of desaturase and hydrogenase enzymes [15,16].

Despite advances in the technological process, these formulas still present great differences in composition when compared to human milk [13]. The main fatty acids present in vegetable oils are oleic (OA), linoleic (LA) and linolenic acid (LAA), but the presence of these nutrients in infant formulas does not ensure the adequate intake of α-linolenic acid and long-chain fatty acid derivatives (EPA, DHA and ARA).

In literature there are few studies in which the lipid profile of infant formulas indicated for infants was evaluated. Therefore, it is of paramount importance to carry out further studies to quantify the lipid fraction of infant formulas, considering their nutritional and physiological value. The present study thus sought to determine the lipid content and lipid profile of different infant formulas and to compare such data with human milk.

## Materials and methods

### Experimental design and samples

Ten infant formulas with distinct characteristics were analyzed, obtained from a maternal-infant hospital unit. Three different batches of each infant formula were evaluated (Tables 1 and 2). Samples from each batch were analyzed in triplicate.

**Table 1:** Information stated on the labeling of infant formula for infants analyzed.

Classification	Product	Source lipidic *	g/100mL	g/100kcal
Infant formula for infants	RFI	Lipid Profile: 80 g/100g of vegetable fat (rapeseed oil, sunflower, palm, and coconut oil) and 20 g/100g milk fat.	Lipids—3.50 C18:2–0.60 C18:3–0.05 ω3:ω6–10.25	Lipids—5.20 C18:2–0.90 C18:3–0.08 ω3:ω6–10.25
	StIF	Lipid Profile: 97 g/100g of vegetable fat (palm olein, palm kernel oil, canola oil, corn oil, fish oil (a source of DHA), soy lecithin, Mortierella alpina oil (ARA source) and 3 g/100g of milk fat and fish oil, soy lecithin, arachidonic fatty acid.	Lipids—3.60 C18:2–0.50 C18:3–0.067 ω3:ω6–7.9	Lipids– 5.4 C18:2–0.80 C18:3–0.100 ω3:ω6–7.9
	IFPLWN	Lipid Profile: 94 g/100g Vegetable fat (palm, coconut, rapeseed, sunflower, evening primrose and structured oils) (medium chain triglycerides and Mortierella alpina oil) and 6 g/100g animal fat (egg yolk phospholipids, oil fish and milk fat, adding AA at a concentration of 0.46 g/100g DHA and at a concentration of 0.35 g/100g of total lipids.	Lipids—4.40 arachidonic acid.– 0.019 Docosahexaenoic acid– 0.015 C18:2–0.56 C18:3–0.08 ω3:ω6–5.80	Lipids– 5.50 arachidonic acid.– 0.024 Docosahexaenoic acid– 0.018 C18:2–0.70 C18:3–0.10 ω3:ω6–5.80
	IFTPGS	Lipid Profile: 97 g/100g of vegetable fat (palm olein, canola oil, palm oil, corn oil, soy lecithin) and 3 g/100g of milk fat; It provides the recommended levels of linoleic and α-linolenic acids.	Lipids—3.10 C18:2–0.50 C18:3–0.061 ω3:ω6–8.00	Lipids -4.70 C18:2–0.70 C18:3–0.092 ω3:ω6–8.00

* Withdrawal of the information provided by the manufacturer

RIF—Routine Infant Formula; StIF—Starter Infant Formula; IFPLWN—Infant Formula for Premature and Low Weight Newborns; IFTPGS—Infant Formula Thickened with Pregelatinized Starch.

**Table 2:** Information stated on the labeling of segment infant formula and infant formulas for breastfeeding aimed at needs specific dietary analyzed.

Classification	Product	Source lipidic*	g/100mL	g/100kcal
Segment infant formula	SIF	Lipid Profile: 98 g/100g of vegetable fat (palm olein, palm kernel oil, canola oil, corn oil, soy lecithin); 2 g/100g milk fat; It provides the recommended levels of linoleic and α-linolenic acids.	Lipids—3.10 C18:2–0.50 C18:3–0.06 ω3:ω6–7.7	Lipids– 4.60 C18:2–0.70 C18:3–0.09 ω3:ω6
Infant formulas for breastfeeding aimed at needs specific dietary	IFSACMPM	Lipid profile: LCPUFAs (DHA and ARA, 1.1); medium chain triglycerides.	Lipids– 3.45 Saturated FA– 1.10 Monounsaturated FA– 1.70 Polyunsaturated FA– 0.70 C18:2–0.600 C18:3–0.060 ω3:ω6–10	Lipids– 3.83 Saturated FA * Monounsaturated FA* Polyunsaturated FA* C18:2* C18:3* ω3:ω6–10
	LFIF	Lipid Profile: 98 g/100g vegetable fat (palm olein, canola oil, coconut oil, sunflower oil, soy lecithin, docosahexaenoic acid, arachidonic acid); 2 g/100g milk fat. Soya lecithin addition, docahexanoico acid, arachidonic acid.	Lipids– 3.30 C18:2–0.50 C18:3–0.067 ω3:ω6–8.00	Lipids– 5.00 C18:2–0.80 C18:3–0.099 ω3:ω6–8.00
	SPIIF	Lipid Profile: 100 g/100g vegetable fat (palm olein, soy oil, coconut oil, sunflower oil); It provides the recommended levels of linoleic and α-linolenic acids.	Lipids– 3.40 C18:2–0.60 C18:3–0.066 ω3:ω6–9.2	Lipids– 5.40 C18:2–0.90 C18:3–0.098 ω3:ω6–9.2
	WPEHIF	Lipid profile: 50 g/100g medium-chain triglycerides; 49% vegetable oils (rapeseed, sunflower, palm); 1% fish oil and Mortierella alpina oil; adding ARA (0.2 g/100g) and DHA (0.2 g/100g) of total lipids.	Lipids– 3.50 Arachidonic acid– 0.0067 Docosahexaenoic acid– 0.0067 C18:2–0.48 C18:3–0.09 ω3:ω6–5.4	Lipids– 5.30 Arachidonic acid– 0.010 Docosahexaenoic acid– 0.010 C18:2–0.72 C18:3–0.13 ω3:ω6–5.4
	WPPHIF	Lipid Profile: 97 g/100g of vegetable fat (palm olein, coconut oil, sunflower oil, fish oil—a source of DHA, vegetable oil from Mortierella alpina—ARA source); 3 g/100g milk fat; It provides the recommended levels of linoleic and α-linolenic acids. Adding arachidonic fatty acid.	Lipids– 3.40 C18:2–0.50 C18:3–0.052 ω3:ω6–9.50	Lipids– 5.10 C18:2–0.70 C18:3–0.078 ω3:ω6–9.50

* Withdrawal of the information provided by the manufacturer

SIF—Segment Infant Formula; IFSACMPM—Elementary Infant Formula for Severe Allergy to Cow's Milk Proteins and Multiple Foods; LFIF—Lactose-Free Infant Formula; SPIIF—Soy Protein Isolate-based Infant Formula; WPEHIF—Whey Protein Extensively Hydrolyzed Infant Formula; WPPHIF—Whey Protein Partially Hydrolyzed Infant Formula.

Regarding human milk, samples were collected from 10 donors from the milk bank of the University Hospital of Brasilia (UHB). The donors had a mean age of 26 years, mean body mass of 72 kg, height ranging from 1.50 to 1.68 m and lactation period between 30 and 60 days postpartum, so that the milk could be considered mature at the time of collection. The gestational period of nine mothers corresponded to the range between 38 and 41 weeks. Only one of the mothers presented a premature gestational period, equal to 34 weeks. All human milk samples were analyzed separately in their original form. Infant formulas were mixed according to the manufacturer's instructions on the label (4.4 g of powder in 30 mL of water). This study was approved by the National Commission for Ethics in Research (NCER), according to the Ethics Assessment Presentation Certificate (EAPC) n° 44750415.6.0000.5558, as per the legislation in force in Brazil. It should be emphasized that during the research there was no direct contact with the donors, which is why the Research Ethics Committee did not require completion and signing of the Informed Consent Form (ICF).

### Chemical analysis of infant formula human milk

#### Total lipids

The extraction of total lipids was performed by the Gerber—Van Gulik butyrometric method and the fat content read directly on a butyrometer rod in g/100g [17,18,19,20]. For the infant formula samples, total lipid extraction was performed by the butyrometer method [21], in triplicate.

#### Esterification chromatography of fatty acids esterified

For methylation lipid aliquots of 20 mg were taken and transferred to a test tube with screw cap; 1.5 mL of 0.5 N potassium hydroxide in methanol was then added, followed by vortexing for 1 minute and heating in a water bath at 70°C for 5 minutes, then cooled immediately under running water. After cooling, 2 mL of 12% (w/w) BF_3_ in methanol were added and the samples were vortexed again for 1 minute. Then, the tube was heated in a water bath at 70°C for 5 minutes and immediately cooled in water [22]. Subsequently, 2.5 mL of saturated NaCl and 1 mL of hexane were added to the mixture, followed by centrifugation for 10 minutes at 1200 rpm. The supernatant was collected and transferred to a 2 mL volumetric capacity glass tube with screw cap, aluminum septum and saturated nitrogen atmosphere. The samples were stored in a freezer at -18°C until the time of analysis by chromatography [23].

The analysis of esterified fatty acids was performed on a GC-2010 Shimadzu gas chromatograph with MS-QP2010 Plus detector (quadrupole, electron impact) and AOC-5000 autoinjector. Separation of the fatty acids was performed using the J & W Scientific 122–2362 DB-23 column with dimensions of 60 m (length), 0.25 mm ID (internal diameter) and 0.25 μm (film thickness). The chromatographic conditions established were: Split type injection with injector temperature equal to 260°C; the column heating ramp was programmed to start at 140°C, remaining at this temperature for 5 minutes, then set to increase by 2°C every minute until reaching 240°C at the end of the 56 minute chromatographic run. Helium was used as the carrier gas, with continuous flow in the column of 0.40 mL/minute. The interface temperature of the MS-QP2010 Plus detector was 260°C, and the injected sample volume was 1 μL.

The identification of each fatty acid was performed by comparison with the retention time of the standard fatty acids of the Supelco 37 component FAME mix (Supelco^®^, USA) and confirmed with the spectra of such substances, already existing in a library in the program of the equipment itself. The results were expressed as a percentage of the area of each fatty acid, in relation to the area of total fatty acids.

### Statistical analysis

The experiment to determine the fatty acid profile was conducted in a randomized block design, with 11 treatments, three blocks and in triplicate. Each of the 10 infant formulas corresponded to a treatment. The treatment corresponding to human milk was used for comparison with data on the infant formulas. Initially, lipid content and lipid profile data were submitted to analysis of variance (p<0.05). When there was a significant difference, the Tukey test was performed at 5% probability, using the Assistat 7.6 software.

## Results

Regarding human milk, we observed that there was a significant difference between values related to the lipid fraction in infant formulas, with a variation between 2.60 and 4.27 g/100g (p<0.05) (Table 3). The mean values for the lipid fraction in the Routine Infant Formula (RIF) and in the Whey Protein Partially Hydrolyzed Infant Formula (WPPHIF), equivalent to 4.23 and 4.27 g/100g, were higher than the value obtained for human milk. The Segment Infant Formula (SIF) and Infant Formula for Premature and Low Weight Newborns (IFPLWN) had mean values of 2.60 and 2.77 g/100g, lower than that of human milk. The total lipid contents of the infant formulas IFPLWN and SIF were significantly different (p<0.05) from the values obtained for the other infant formulas.

**Table 3:** Content of lipids expressed in g / 100ml and g / 100kcal in infant formula and human milk.

Classification	Infant formulas	Lipid content (g/100ml)	Lipid content (g/100 kcal)
Infant formula for infants	RFI	4.23±0.21 a	6.30
	StIF	3.95±0.05 ab	5.88
	IFPLWN	2.77±0.35 cd	3.46
	IFTPGS	3.50±0.26 b	5.24
Segment infant formula	SIF	2.60±0.17 d	3.83
Infant formulas for breastfeeding aimed at needs specific dietary	IFSACMPM	4.10±0.10 ab	5.75
	LFIF	3.93±0.06 ab	5.91
	SPIIF	4.06±0.51 ab	6.02
	WPEHIF	3.43±0.06 bc	5.20
	WPPHIF	4.27±0.06 a	6.33
Human Milk		3.44±0.50 bc	

RIF—Routine Infant Formula; StIF—Starter Infant Formula; IFPLWN—Infant Formula for Premature and Low Weight Newborns; IFTPGS—Infant Formula Thickened with Pregelatinized Starch; SIF—Segment Infant Formula; IFSACMPM—Elementary Infant Formula for Severe Allergy to Cow's Milk Proteins and Multiple Foods; LFIF—Lactose-Free Infant Formula; SPIIF—Soy Protein Isolate-based Infant Formula; WPEHIF—Whey Protein Extensively Hydrolyzed Infant Formula; WPPHIF—Whey Protein Partially Hydrolyzed Infant Formula.

Means followed by the same letter in the column do not differ by Tukey test at 5% probability.

Calculation made from the energy value declared on the product label.

We also found that there was a significant difference (p<0.05) in relation to human milk for capric acid (C10:0) in the Whey Protein Extensively Hydrolyzed Infant Formula (WPEHIF), for myristic acid (C14:0) in the Elementary Infant Formula for Severe Allergy to Cow's Milk Proteins and Multiple Foods (IFSACMPM) and in the WPEHIF, and for palmitic acid (C16:0) in the infant formulas IFPLWN, IFSACMPM, WPEHIF and WPPHIF (Table 4). Regarding stearic acid, all the infant formulas analyzed differed significantly from human milk. The WPEHIF, IFSACMPM and IFPLWN formulas stood out as having the highest mean values, equivalent to 13.67, 7.21 and 4.32 g/100g, respectively. For capric acid (C10:0), the infant formula WPEHIF had the highest average value, equivalent to 13.81 g/100g. The sum of the mean values of caprylic and capric acids corresponded to 27.48 and 14.84 g/100g of fatty acids present in the infant formulas WPEHIF and IFSACMPM. The infant formulas WPEHIF and IFSACMPM presented the lowest mean values for the lauric acid fractions (C12:0) and myristic acid fractions (C14:0). As for palmitic acid (C16:0), the Lactose-Free Infant Formula (LFIF), Soy Protein Isolate-based Infant Formula (SPIIF), Infant Formula Thickened with Pregelatinized Starch (IFTPGS) and SIF had average values equivalent to 22.10, 19.88, 19.53 and 20.79 g/100g, statistically equal (p>0.05) to that of human milk.

**Table 4:** Percentage of saturated fatty acids in infant formula and human milk.

Fatty acids	Infant formulas–(g/100g)										Human Milk
	RFI	StIF	IFPLWN	IFTPGS	SIF	IFSACMPM	LFIF	SPIIF	WPEHIF	WPPHIF	
C 8:0	0.87±0.51b	0.68±0.34b	4.32±1.03b	0.82±0.72b	0.15±0.13b	7.21±6.31ab	0.63±0.31b	1.63±0.09b	13.67±7.82a	1.05±1.22b	ND
C 10:0	0.76±0.72b	2.68±2.16b	3.83±0.95b	0.82±0.71b	0.40±0.18b	7.63±6.17ab	0.66±0.40b	1.42±0.19b	13.81±6.99a	1.22±0.83b	1.37±0.71b
C 12:0	5.95±1.53ab	8.55±3.15ab	10.96±0.89ab	17.32±9.98a	9.90±0.16ab	3.29±5.17b	8.39±0.85ab	12.36±3.11ab	2.03±3.42b	11.36±2.19ab	6.41±2.17ab
C 14:0	4.11±1.60abc	4.38±1.71abc	5.53±0.20abc	4.40±1.22abc	4.12±0.26abc	1.69±2.58bc	4.39±0.62abc	5.07±0.40abc	1.27±1.93c	5.85±0.99ab	6.17±2.33a
C 16:0	16.02±1.75bcd	19.18±1.72abc	14.53±0.38cd	19.53±2.02abc	20.79±0.33ab	8.18±1.99e	22.10±0.40a	19.88±0.84 abc	12.02±4.93de	10.47±0.12de	20.19±1.33ab
C 18:0	5.89±1.19b	5.43±0.38b	4.81±0.05b	4.62±0.49b	4.38±0.21b	5.88±1.19b	5.07±0.41b	5.42±2.64b	5.24±1.37b	5.03±0.23b	10.26±1.82a
C 20:0	0.28±0.48a	0.66±0.20a	042±0.07a	0.60±0.16a	0.25±0.07a	0.31±0.38a	0.27±0.28a	0.56±0.26a	0.63±0.10a	0.20±0.35a	0.14±0.21a
C 21:0	ND	0.24 ± 0.34a	ND	ND	ND	ND	ND	ND	ND	ND	0.89 ± 0.49a
C 22:0	0.29±0.27ab	0.50±0.28ab	0.42±0.20ab	0.17±0.15ab	ND	0.66±0.59ab	0.09±0.08b	0.13±0.18b	0.23±0.25ab	0.82±0.20a	ND
Total	34.16	42.30	44.82	48.27	39.99	34.84	41.60	46.46	48.91	36.01	45.42

RIF—Routine Infant Formula; StIF—Starter Infant Formula; IFPLWN—Infant Formula for Premature and Low Weight Newborns; IFTPGS—Infant Formula Thickened with Pregelatinized Starch; SIF—Segment Infant Formula; IFSACMPM—Elementary Infant Formula for Severe Allergy to Cow's Milk Proteins and Multiple Foods; LFIF—Lactose-Free Infant Formula; SPIIF—Soy Protein Isolate-based Infant Formula; WPEHIF—Whey Protein Extensively Hydrolyzed Infant Formula; WPPHIF—Whey Protein Partially Hydrolyzed Infant Formula.

C 8:0 –Caprylic acid; C 10:0 –Capric acid; C 12:0 –Lauric acid; C 14:0 –Myristic acid; C 16:0 –Palmitic acid; C 18:0 –Stearic acid; C 20:0 –Arachidic acid; C 21:0 –Heneicosanoic acid; C 22:0 –Behenic acid; C 16:1 –Palmitoleic acid; C 18:1 –Oleic acid; C 18:2 –Linoleic acid; C 18:3 –Linolenic acid; C 20:1 –Eicosanoic acid.

ND—not detected by the method.

Means followed by the same letter in the column do not differ by Tukey test at 5% probability.

For the stearic acid fraction (C18:0), we identified that all infant formula samples were significantly equal to each other (p>0.05), but significantly different (p<0.05) with respect to human milk. The percentage of stearic acid ranged from 4.38 to 5.89 g/100g; in human milk the percent value determined was 10.26 g/100g. Regarding arachidic acid (C20:0), there was no significant difference (p>0.05) between the mean values obtained in infant formulas and in human milk, where in general the values were between 0.20 and 0.66 g/100g. Heneicosanoic acid (C21:0) was detected only in the Starter Infant Formula (StIF) and in human milk, and were significantly the same. Behenic acid (C22:0) was not detected in the infant formula SIF, nor in human milk. The mean percentage values were between 0.08 and 0.82 g/100g. The LFIF and SPIIF infant formulas differed statically from the others (Table 4).

With regards to the monounsaturated fatty acid profile (Table 5), when compared to human milk all infant formulas analyzed statistically differed in terms of the palmitoleic acid fraction (C16:1) (Table 5). When compared to each other, the infant formulas were significantly equal. Human milk presented the highest mean value of this nutrient, equivalent to 3.73 g/100g (p<0.05), while in infant formulas the mean values remained between 0.03 and 0.26 g/100g. Only in the infant formula SIF was palmitoleic acid not detected. For the oleic acid fraction (C18: 1), only the infantile formulas RIF, IFSACMPM and WPPHIF showed significant differences (p<0.05) in relation to human milk and when compared to the other infant formulas.

**Table 5:** Percentage of monounsaturated and polyunsaturated fatty acids in infant formula and human milk.

Fatty Acids	Infant formulas—(g/100g)										Human Milk
	RFI	StIF	IFPLWN	IFTPGS	SIF	IFSACMPM	LFIF	SPIIF	WPEHIF	WPPHIF	
C 16:1	0.20±0.35b	0.26±0.05b	0.23±0.03b	0.15±0.13b	ND	0.07±0.13b	0.04±0.07b	0.25±0.12b	0.03±0.05b	0.07±0.13b	3.73±1.37a
C 18:1	45.52±8.12a	38.16±9.04ab	38.44±2.38ab	34.42±7.97ab	40.06±1.16ab	43.93±8.44a	36.86±2.15ab	32.32±3.99ab	30.18±2.61b	43.95±4.71a	27.94±2.29b
C 18:2	19.81±0.84a	18.74±2.54a	15.89±0.49a	16.65±3.02a	19.84±0.53a	20.87±2.80a	21.15±0.32a	20.71±2.40a	20.07±2.51a	19.46±1.53a	21.44±3.63a
C 18:3	0.32±0.55a	0.54±0.27a	0.61±0.05a	0.40±0.35a	0.12±0.10a	0.29±0.50a	0.35±0.30a	0.27±0.02a	0.82±0.36a	0.52±0.26a	0.58±0.38a
C 20:1	ND	ND	ND	0.10±0.18a	ND	ND	ND	ND	ND	ND	0.89±0.29a
Total (AGMI)	45.72	38.42	38.67	34.67	40.06	44.00	36.90	32.57	30.21	44.02	32.56
Total (AGPI)	20.13	19.28	16.50	17.05	19.96	21.16	21.50	20.98	20.89	19.98	22.02
Total	65.85	57.70	55.17	51.72	60.02	65.16	58.40	53.55	51.10	64.00	54.58

RIF—Routine Infant Formula; StIF—Starter Infant Formula; IFPLWN—Infant Formula for Premature and Low Weight Newborns; IFTPGS—Infant Formula Thickened with Pregelatinized Starch; SIF—Segment Infant Formula; IFSACMPM—Elementary Infant Formula for Severe Allergy to Cow's Milk Proteins and Multiple Foods; LFIF—Lactose-Free Infant Formula; SPIIF—Soy Protein Isolate-based Infant Formula; WPEHIF—Whey Protein Extensively Hydrolyzed Infant Formula; WPPHIF—Whey Protein Partially Hydrolyzed Infant Formula.

Means followed by the same letter on the line do not differ by Tukey test at 5% probability.

C16: 1—Palmitoleic acid; C18: 1—Oleic acid; C 18: 2—Linoleic acid; C 18: 3—Linolenic acid; C 20: 1—Eicosanoic acid.

ND—not detected by the method.

For the infant formulas we detected percent values greater than 30.18 g/100g for oleic acid (C18:1). In the infant formulas RIF, WPPHIF and IFSACMPM the mean values were equal to 45.52, 43.95 and 43.83 g/100g, statistically superior (p<0.05) to that obtained in human milk (27.94 g/100g). Regarding linoleic acid (C18:2), there was no significant difference among the values obtained for the different infant formulas and in human milk (p>0.05); they varied between 15.89 and 21.15 g/100g and human milk was in the same range (21.44 g/100g). There was also no significant difference (p>0.05) for the linolenic acid fraction (C18:3) in relation to human milk and among the infant formulas analyzed. The mean percentage values were between 0.12 and 0.82 g/100g. Eicosanoic acid was quantifiable only in the IFTPGS infant formula and human milk, with mean percent values equivalent to 0.10 and 0.89 g/100g, which were significantly equal (p>0.05) (Table 5).

In all infant formulas analyzed, the sum of the mean values for unsaturated fatty acids was higher than 51.00 g/100g. The sum of the mean percentage values for the fractions of unsaturated fatty acids of the infantile formulas RIF, IFSACMPM and SIF were 65.85, 65.16 and 60.02 g/100g, respectively. In human milk, the sum of the mean values for unsaturated fatty acids was 54.58 g/100g. We also observed that in the infantile formulas WPEHIF and SPIIF the polyunsaturated fatty acids represented 40.88 and 39.18 g/100g of the total unsaturated fatty acids, respectively. In human milk, polyunsaturated fatty acids represented 40.34 g/100g of the total unsaturated fatty acids.

## Discussion

We identified in this study that 40% (n = 4) and 20% (n = 2) of infant formulas showed a significant difference in relation to the percentage of total lipids in human milk and among the other infant formulas analyzed. We also observed a significant difference in relation to the fatty acid profile, both in relation to human milk and among the infant formulas analyzed. In part, these results are justified since the products were obtained by a mixture of lipids from vegetable and animal origin, or only of vegetal origin, with variable ingredients in different proportions (Tables 1 and 2). The infant formula industry seeks primarily to provide adequate levels of essential fatty acids—linoleic and α-linolenic acids [24].

Thus, it is most relevant to identify if the products available on the market meet the nutritional requirements described in literature, since these nutrients are the main source of energy for the infant, are fundamental for the absorption of the fat-soluble vitamins, for the composition of the cell membranes and for providing structural components to the retina and brain, in addition to the fact that some fatty acids play a determinant role in the expression of some genes [25].

There is little information in literature on the total lipid content of infant formulas. Riva *et al*. [26] analyzed the composition of 30 different infant formulas available on the Italian market based on information obtained from the product label and identified a variation between 2.9 g/100mL (4.32 g/100kcal) and 3.8 g/100mL (5 g/100kcal) for the lipid content. Kus *et al*. [27] determined the lipid content in 14 infant formula samples and the data obtained was in agreement with that transcribed in the labels of these samples. Rêgo *et al*. [25] performed an assessment of infant formulas available on the Portuguese market (n = 87) and identified that the total lipid fraction ranged from 4.7 g/100 kcal to 5.3 g/100 kcal, while for human milk the reference value was 5.6 g/100 kcal. Zunin *et al*. [28] analyzed 32 infant formula samples and identified a variation between 22.9 and 30.1 g/100g, dry basis, for the total lipid fraction.

According to The European Society for Pediatric Gastroenterology Hepatology and Nutrition (ESPGHAN), the minimum total lipid content in infant formulas should be 4.4 g/100 kcal (1.05 g/100 kJ) and the maximum 6.0 g/100 kcal (1.4 g/100 kJ) [29]. In this study, two infant formulas—IFPLWN and SIF—had a reduction of 43% and 36%, while infantile formulas RIF and WPPHIF showed an increase of around 5% in the total lipid content expressed in g/100kcal [29]. However, maximum and minimum lipid intake values, physiologically tolerable for the well-being of infants, are not well defined [30].

Regarding the total lipid content in human milk, it is known that in general the lipid concentration is generally lower in colostrum than in mature milk and the lipid content of normal milk increases progressively during nursing; however, the triacylglycerol fraction does not undergo major changes between the lactation stages [8,31,32,33]. Studies have shown that the total lipid content of human milk tripled, on average, from 2.4 to 7.5 g/100 mL during breastfeeding [34]; and that with the advancement of lactation the lipid content increased by 2% three days after delivery in the following order: colostrum, transition milk, mature milk. After 3 months, the increase was greater than 4% [35]. In general, the lipid content in human milk ranged from 3 to 5 g/100 mL [1].

We found that for the infant formulas and human milk samples, the data was partially in agreement with the information stated on the product labels and with literature [1,8,36]. The lipid fraction of human milk should contribute 40% to 55% of the total energy consumed by the infant, and also contribute to the other physiological processes responsible for health of the infant; and therefore lower lipid contents may favor the supply of reduced concentrations of essential fatty acids and provide lower caloric intake [1,2,8].

Regarding the profile of saturated fatty acids, we verified that the values obtained for the infant formulas were in accordance with literature, which highlights a variation of 28.70 to 48.99 g/100g for the fraction of saturated fatty acids in human milk [8,37]. Palmitic acid was the predominant fatty acid, except for the formulas IFSACMPM and WPPHIF, and was in significantly equal concentration when compared to human milk (Table 4).

Research showed variations between 17.96 and 27.42 g/100g for palmitic acid in 11 infant formulas, and an average content of 19.48 g/100g in human milk. The authors further reported that palmitic acid significantly decreased from the colostrum stage to the mature milk stage [37]. Another study with samples of human milk collected in different countries showed a variation between 17.30 and 25.14 g/100g for this fatty acid [8]. These variations may be due to the lactation stage, individual factors of nursing mothers, adiposity, eating habits and others [38]. The presence of palmitic acid (C16:0) in human milk ensures greater digestibility and use of this nutrient as an energy source, generating other fatty acids, and can also be stored by the infant. Moreover, because it is not essential, palmitic acid can also be synthesized by the human organism [1,8,23,39].

However, unlike human milk, the lipid fraction of infant formulas is mainly composed of oils of vegetable origin [8]. Palmitic acid is therefore predominantly esterified in the *sn-1* (or α) and *sn-3* (or α’) positions, and thus free palmitic acid binds calcium and becomes insoluble in the intestine so that it is not absorbed, and is eliminated in the feces [23].

This is because in human milk esterification occurs at the *sn-2* (or β) position of the triglyceride, allowing pancreatic lipases to selectively hydrolyze fatty acids at the *sn-1* and *sn-3* positions producing *sn-2* monoacylglycerols and free fatty acids, which are absorbed, re-esterified and secreted in the plasma. Approximately 60% of all palmitic acid (C16:0) in human milk is in the *sn-2* position of the triacylglycerol [8]. This is due to the higher polarity and solubility in water of monoacylglycerol with palmitic acid in the *sn-2* position relative to its free form [1,2,30,40].

Research conducted to evaluate the percent of fatty acids in the *sn-2* position in human milk samples and infant formulas showed 7.33 to 62.33 g/100g of palmitic acid in the infant formulas was in the *sn-2* position, while in human milk 87.86 g/100g was in the *sn-2* position [37]. Straarup *et al*. [14] described the composition and stereospecificity of fatty acids in 28 infant formulas and verified that the percentage of palmitic acid in the *sn-2* position ranged from 0.4 to 10.1 g/100g.

The second fraction, quantitatively more important, was that of lauric acid (C12:0) and is in accordance with values described in literature [8,37]. As for the sum of lauric (C12:0) and myristic acids (C14:0), we found that only the infant formula IFTPGS showed a sum of values higher than 20 g/100g of the total fatty acid content (Table 4); the others were in accordance with the recommendations of ESPGHAN [29]. Research carried out in Italy with 32 infant formulas and in Spain with 11 infant formulas identified that the sum of the percentages of these fatty acids was less than 20 g/100g of total fatty acids [28,37].

The percentage of fatty acids with hypercholesterolemic potential, corresponding to the sum of the lauric acid (C12: 0), myristic acid (C14: 0) and palmitic acid (C16: 0) fractions in the infant formulas analyzed, was in agreement with literature as well as with other infant formulas and human milk [1,27,37,39]. Saturated fat (C12:0, C14:0 and C16:0) raises the plasma cholesterol concentration, especially C14:0, whereas C18:0 has a neutral effect on cholesterol [1,41]. When consumed in excess, saturated fatty acids can increase the risk of developing heart disease. Carlson *et al*. [42] reported that lauric acid and myristic acid (C14:0) are potentially more cholesterolemic.

With respect to stearic acid (C18:0), we confirmed that the values obtained for both the infant formulas and human milk were lower than those reported in literature [8,37]. This fatty acid (C18:0) is generally found at more moderate levels in relation to palmitic acid, and in human tissue this component is rapidly converted to oleic acid (C18:1) [1].

It is possible to justify the data for the fractions of palmitic, lauric and myristic acids, especially since the composition of each product (Tables 1 and 2) showed that the lipid fraction of the infant formulas analyzed was composed of oils of vegetable origin (palm and coconut oils), rich in these nutrients [43]. On the other hand, medium-chain triglycerides (MCT) may have contributed to the average values (g/100g) of caprylic acid (C8:0) in the infant formulas samples IFPLWN (4.32 g/100g), IFSACMPM (7.21 g/100 g) and WPEHIF (13.81 g/100g), since MCT molecules are formed by three saturated fatty acids containing 6 to 12 carbon atoms, esterified to glycerol, including caproic acid (C6:0), caprylic acid (C8:0), capric acid (C10:0) and lauric acid (C12:0) [44]. This fatty acid fraction was not detected in human milk samples. It is interesting to observe that the sources of lipids of vegetable origin vary very little among infant formulas, leaving the manufacturer of each formulation to justify the different fatty acid profiles determined in the samples analyzed.

Regarding the sum of total monounsaturated fatty acids—palmitoleic (C16:1), oleic (C18:1) and eicosanoic (C20:1), the results obtained in this study are in agreement with those in literature for human milk [8]. The oleic acid fraction was the largest among all samples of infant formulas analyzed, partially agreeing with literature data for both infant formulas and human milk (Table 5) [8, 37]. In addition to providing energy reserves, among other functions, oleic acid controls the synthesis of medium chain fatty acids and reduces the melting point of triglycerides, providing the fluidity necessary for formation, transport and metabolism of fat globules. Monounsaturated fatty acids can be synthesized in the body. It is also known that due to the various benefits of this nutrient fraction, reports suggest the consumption of vegetable oils rich in these compounds by mothers [8,45,46].

With respect to essential fatty acids, the data obtained in this study indicated that all infant formula samples were significantly equally to each other and to human milk. Straarup *et al*. analyzed 28 infant formulas and obtained the following results for the fractions of linoleic (C18:2n-6) and linolenic fatty acids (C18:3n-3), respectively: 11.9 to 20.5 g/100g and 0.9 to 2.2 g/100g for products (n = 5) intended for preterm infants; 5.3 to 26.0 g/100g and 0.3 to 2.0 g/100g for follow-up formulas (n = 5) and for full-term infants; and 5.3 to 26.0 g/100g and 0.3 to 2.0 g/100g for infant formulas (n = 11) for specific dietary needs. López-López et al. [37] analyzed 11 infant formulas and identified the following data: 8.93 to 18.43 g/100g for the linoleic acid fraction (C:18-2n-6) and 0.67 to 2.83 g/100g for the linolenic acid fraction (C18:3n-3). As for variation of these fatty acids in the human milk samples, the value obtained for the linoleic acid fraction (C18:2n-6) was 21.44 g/100g, in agreement with the variation ranges presented in literature: 10 to 25 g/100g.

Thus, the data obtained for the infant formula samples analyzed in this study tend to present similar behavior regarding the fraction of linoleic acid (C18:2n-6), and data lower than that found in literature for the linolenic acid fraction (C:18-3n-3). The fraction of linoleic acid (C18:2n-6) in infant formulas, as in human milk, should contribute about 7 to 20 g/100g of total fatty acids, sufficient to cover the minimum requirements for this nutrient [29,30]. Elevated concentrations of this fatty acid may induce undesirable metabolic effects in relation to the lipoprotein metabolism, immune function, eicosanoid balance and oxidative stress.

In relation to the fraction of linolenic acid (C18:3n-9), we detected that the value obtained was lower than the recommended value (1% of total fatty acids) in human milk and in the different infant formulas. It is emphasized that linolenic acid can increase the risk of lipid oxidation producing rancidification and adverse effects on stability of the infant formula [1,29]. Other authors reported values between 0.49 and 1.72 g/100g [8] and 1.4 g/100g [47] in human milk, and from 0.9 to 2.4 g/100g (C18:3n-3) [14] in different infant formulas.

Although the sum of monounsaturated fatty acids and polyunsaturated fatty acids, for the infant formulas and human milk samples, is partially in agreement with literature [8], ESPGGHAN [29] does not define the desirable ratio for the saturated, monounsaturated and polyunsaturated fatty acid fractions. This makes it possible to speculate whether the concentration of saturated fatty acids may be close to that of human milk or could be lower. In human milk, the ratio of saturated, monounsaturated and polyunsaturated fatty acids is 45:40:15. In this study, the infant formulas StIF (42.30:38.42:19.28) and IFPLWN (44.82:38.67:16.50) were the closest to this proportion.

It was also found that for the infant formula samples analyzed, the values obtained for the n-6:n-3 ratio were not in accordance with the recommendations of ESPGHAN [29]. Straarup et al. [14] found a similar response to that of our research, while studies performed by López-López et al. [37], Riva *et al*. [26] and Kus et al. [27] showed compliance with the ESPGHAN recommendations regarding the n-6:n-3 ratio.

Literature recommends a ratio of linoleic acid (C18:2n-6) to linolenic acid (C18:3n-3) of 5 to 15, since it is known that the fatty acid series (n-3, n-6, n-7 and n- 9) compete with one another for the metabolic pathways of stretching and desaturation, and this harmony is important for proper balance in the production of long chain polyunsaturated fatty acids—ARA, DHA, EPA—and proper functioning of the organism [8,26,48].

Thus, the n-6/n-3 ratio is an important indicator of the nutritional quality of infant formulas [8,49]. Human milk has an n-6/n-3 (5:1–10:1) ratio that prevents both excess linoleic acid and reduced DHA synthesis [26].

Essential fatty acids are precursors of n-3 and n-6 long-chain polyunsaturated fatty acids, which yield arachidonic acid—ARA (20:4 n-6), eicosapentaenoic acid—EPA (20:5 n-3) and docosahexaenoic acid—DHA (22:6 n-3) [50]. Arachidonic acid is the most important metabolite of linoleic acid in animal tissues, both quantitatively and biologically. Generally, it is the most abundant polyunsaturated fatty acid in the fraction of phospholipids and a precursor of several families of eicosanoids, such as series 2 prostaglandins (PG_2_), thromboxanes, leukotrienes and lipoxins (anti-inflammatory mediators) [18,51]. Arachidonic and docosahexaenoic fatty acids are the main components of the phospholipid membrane in the cell, and are the predominant polyunsaturated fatty acids in the central nervous system. Docosahexaenoic acid is the most abundant fatty acid in the retinal photoreceptor membrane [50].

Colostrum is richer in unsaturated fatty acids than mature milk, and these are also found in higher concentration in the colostrum and mature milk of mothers of preterm infants. However, the concentration of long-chain polyunsaturated fatty acids in human milk declines rapidly after three months postpartum [4].

Considering the available information on the composition of formulas and the fatty acid profile, it is important to observe the contribution of primrose, sunflower, corn and soybean oils on the percentage of linoleic acid in the infant formulas analyzed [43]. Infant formulas are expected to approach the maximum lipid composition of breast milk, since essential fatty acids must be ingested in the diet. The label information requirement for these products is relatively recent, and therefore we identified omission on the labels of products with regards to information on the content of these fatty acids. Above all, it is necessary that the food industry adjust the lipid profile of infant formulas in such a way that these products become equivalent to breast milk.

### Limitation of the study

It is important to highlight the need to determine the stereospecific structure of palmitic acid (16:0) in the different infant formulas in future studies, considering that most of this fatty acid present in human milk is located in the *sn-2* position of the triacylglycerol molecule, in contrast to cow's milk and vegetable oils, which contain most of the fatty acid mentioned in the outermost positions of the triglyceride molecules [8,23]. It should also be noted that it was not possible to quantify ARA and DHA, even in the infant formulas whose manufacturers indicated the addition of these fatty acids on the labels. This limitation may be justified by the fact that these are long-chain compounds with a high number of instaurations, and it is not possible to quantify them with a 60 m chromatographic column.

## Conclusions

Despite the observed differences in lipid content and fatty acid profile in infant formulas, the administration of these products is a viable strategy for the development of infants in or not in specific physiological situations. Likewise, it has been observed that it is still necessary to propose compositions of infant formulas that make it possible to obtain products with a higher content of linolenic acid. This fatty acid is essential and fundamental for the production of one of the long-chain polyunsaturated fatty acids, DHA, essential for many neurological functions and which together with cholesterol can act as a modulator of the structure and function of membranes. In the case of the percent of polyunsaturated fatty acids in relation to the total of unsaturated fatty acids, only the analyzed formulas SPIIF and WPEHIF resemble human milk.
